# Molecular population genetics of *Sex-lethal* (*Sxl*) in the *Drosophila melanogaster* species group: a locus that genetically interacts with *Wolbachia pipientis* in *Drosophila melanogaster*

**DOI:** 10.1093/g3journal/jkab197

**Published:** 2021-06-11

**Authors:** Vanessa L Bauer DuMont, Simone L White, Daniel Zinshteyn, Charles F Aquadro

**Affiliations:** Department of Molecular Biology and Genetics, Cornell University, Ithaca, NY 14853, USA

**Keywords:** germline stem cells, *Wolbachia pipientis*, population genetics, natural selection, genetic conflict

## Abstract

*Sex-lethal* (*Sxl*) is the sex determination switch in *Drosophila*, and also plays a critical role in germ-line stem cell daughter differentiation in *Drosophila melanogaster*. Three female-sterile alleles at *Sxl* in *D. melanogaster* were previously shown to genetically interact to varying degrees with the maternally inherited endosymbiont *Wolbachia pipientis.* Given this genetic interaction and *W. pipientis’* ability to manipulate reproduction in *Drosophila*, we carried out a careful study of both the population genetics (within four *Drosophila* species) and molecular evolutionary analysis (across 20 *Drosophila* species) of *Sxl*. Consistent with earlier studies, we find that selective constraint has played a prominent role in *Sxl’s* molecular evolution within *Drosophila*, but we also observe patterns that suggest both episodic bursts of protein evolution and recent positive selection at *Sxl*. The episodic nature of *Sxl’s* protein evolution is discussed in light of its genetic interaction with *W. pipientis*.

## Introduction

Reproductive success is a key fitness trait governed by a plethora of gene regulatory networks. Despite the presumed functional constraint for such critical genes, many of them have been shown to be evolving rapidly due to positive selection at the amino acid level (Clark *et al.* 2006; Chapman 2008; Wong and Rundle 2013; Popovic *et al.* 2014). For some sets of reproductive loci, this observation makes intuitive sense, including loci involved in species-specific gamete recognition and those involved in coevolutionary conflict between the sexes. Interestingly, non-neutral patterns of amino acid evolution have also been detected at loci involved in the differentiation of germ-line stem cells (GSCs) of *Drosophila* (Civetta *et al.* 2006; Bauer DuMont *et al.* 2007;  Langley *et al*. 2012; Pool *et al.* 2012; Choi and Aquadro 2015; Flores *et al.* 2015a). The temporal and spatial expression of these GSC regulating loci does not coincide with that expected for genes influenced by sperm competition, sexual selection, inbreeding avoidance, and gamete recognition (reviewed in Clark *et al.* (2006). 

Several GSC regulating loci have functions outside the germline (*e.g.*, Bell *et al.* 1988; Yi *et al.* 2008; Saito *et al.* 2010; Le Thomas *et al.* 2013) so their signatures of positive selection could be due to non-gametogenic functions. For many others, it is possible that the positive selection is acting directly on gametogenic functions. We have previously hypothesized that interactions with maternally transmitted endosymbionts could be a gametogenesis-specific driver of positive selection (Bauer DuMont *et al.* 2007; Flores *et al.* 2015a, 2015b). One such endosymbiont, *Wolbachia pipientis*, infects an estimated 66% of arthropod species (Hilgenboecker *et al.* 2008) and is an obligate maternally transmitted endosymbiont that has been shown to manipulate host reproduction systems (Werren *et al.* 2008). *W. pipientis* infection has also been shown to have beneficial consequences. For example, infection in *Drosophila melanogaster* conveys greater resistance to viruses (Hedges *et al.* 2008; Teixeira *et al.* 2008; Chrostek *et al.* 2013) and has been shown to increase female fecundity on low and high iron diets relative to uninfected flies (Brownlie *et al.* 2009).

Here, we explore the phylogenetic patterns of positive selection for amino acid diversification at *Sxl*, the sex determination master switch in *Drosophila* development (Bell *et al.* 1988) that also plays a critical role, along with *bag of marbles* (*bam*), in the maturation of cystoblasts during oogenesis (Chau *et al.* 2009). Proper *bam* function is essential for the start of cystoblast differentiation (McKearin and Spradling 1990). Epistasis experiments revealed that *bam* requires *Sxl* activity for proper germline stem cell daughter differentiation and the presence of both proteins is proposed to be responsible for regulating sex-specific gametogenesis (Chau *et al.* 2009, 2012; Shapiro-Kulnane *et al.* 2015).

In addition to genetically interacting with one another, hypomorphic alleles of both proteins have been found to genetically interact with *W. pipientis* infection. Starr and Cline (2002) reported that *W. pipientis* infection partially rescues the female-sterile phenotype caused by three *Sxl* alleles, though to differing degrees for each allele. Similarly, *W. pipientis* infection can mitigate the mutant phenotype of a hypomorphic allelic combination at the *bam* locus (Flores *et al.* 2015b). Starr and Cline (2002) also reported that *W. pipientis* infection did not rescue mutants in three other genes with ovarian tumor phenotypes (*snf^1621^*, *otu^11^*, and *mei-P26^fs1^*). These results suggest a specific, possibly physical, interaction of *W. pipientis* or its gene products with the *Sxl* and *bam* genes or gene products.

Using methods that consider both polymorphism and divergence we and others have previously shown that in *D. melanogaster* and *D. simulans*, the *bam* locus is evolving rapidly at the amino acid level due to positive selection (Civetta *et al.* 2006; Bauer DuMont *et al.* 2007). Phylogenetic methods for detecting selection across a number of *Drosophila* species did not detect evidence of positive selection, suggesting the selection pressures acting on *bam* are episodic. On the other hand, recent studies of the molecular evolution of *Sxl* have focused on the evolution of *Sxl* in the context of this gene having been recruited to also play an additional role in sex determination in the common ancestor of *Drosophila* and *Scaptodrosophila* (Mullon *et al.* 2012;  Zhang *et al*. 2014). Mullon *et al.* (2012) used the maximum likelihood-based phylogenetic methods implemented in PAML (Yang 2007) within and between three families of Diptera (Drosophilidae, Tephritidae, and Muscidae), and detected both a relaxation of functional constraint and positive selection acting across amino acid sites along the lineage leading to *Drosophila*. However, they observe no evidence of positive selection among the 12 *Drosophila* species suggesting that the positive selection they observed was associated with the acquisition of the sex determination function in the common ancestor of *Drosophila* species.

Here, we test for evidence departures from selective neutrality at *Sxl* within the genus *Drosophila* specifically by incorporating sequence polymorphism and divergence data, and test for departures consistent with lineage-specific positive selection. First, we obtained high-quality Sanger sequencing polymorphism data at *Sxl* in the following species: *Drosophila melanogaster*, *D. simulans*, *D. ananassae*, and *D. pseudoobscura*. Except for *D. pseudoobscura*, these species have been reported to be infected with *W. pipientis* (Mateos *et al.* 2006). Second, we searched eight additional sequenced *Drosophila* genomes to annotate *Sxl* orthologs, bringing the total number of *Drosophila Sxl* orthologous sequences to 20 for phylogenetic analysis. The inclusion of polymorphism data and additional *Drosophila* species allowed us greater power to detect potential signatures of positive selection at *Sxl* within the genus *Drosophila*.

## Materials and methods

All DNA was extracted using the Qiagen puregene kit A DNA isolation kits (Qiagen). There are multiple *Sxl* transcripts, due to alternative splicing, but most of them include exons 5 through 8 in *D. melanogaster* according to Flybase.org genome annotations (St Pierre *et al.* 2014). These four exons include roughly 87% of the coding sequence for *Sxl* transcripts. Thus we used PCR primers SxlF1–5′-agcatcgaaatagggatgcg-3′ and SxlR1–5′-aggccttctcacaacactag-3′ to amplify and sequence a 1486 bp fragment from *D. melanogaster* that included exons 5 through 8 in order to focus our analyses on the most commonly used exons, including the location of the *Sxl* hypomorphs rescued by infection by *W. pipientis*. This choice may lead us to miss natural selection acting on unique transcripts, but we feel it does not compromise our focused analysis here. We used Promega GoTaq for amplification following their standard protocol. Sanger sequencing was performed using the PCR primers and internal sequencing primers for each species (primer sequences available upon request) through the Cornell Institute of Biotechnology Genomics Facility (https://www.biotech.cornell.edu/core-facilities-brc/facilities/genomics-facility).

For *D. melanogaster*, we sequenced the 1486 bp fragment including exons 5–8 in 20 extracted X-chromosome lines by crossing a female Fm7a balancer stock with a single male from each of 20 isofemale lines collected in 2012 by Russell Corbett-Detig (see Pool *et al.* 2012) in Siavonga, Zambia Africa. For *D. simulans*, we used 10 isofemale lines from a Madagascar population sample collected in 1998 by J. William O. Ballard. For *D. pseudoobscura*, we included 29 isofemale lines we obtained from Stephen W. Schaeffer, 8 of which were collected by Stephen W. Schaeffer in 2005 from Kaibab National Forest, Arizona and 11 from Mesa Verde National Park, Colorado in 2005, and 10 of which were sent to Steven W. Schaeffer in 2007 by Bryant McAllister that had been collected by Sara Sheeley in 2006 in the Bosque Del Apache National Wildlife Refuge, San Antonio, New Mexico. Our isofemale line of *D. miranda* (stock MSH22) was obtained from Doris Bachtrog in 2013 who obtained it from Mohamed Noor who originally collected it on Mount Saint Helena in California in 1997—Noor *et al.* (1998). Finally, we also surveyed *Sxl* variation across 12 lines of *D. ananassae* collected from Bangkok, Thailand in 2002 by Aparup Das and Uraiwan Arunyawat and provided to us by Wolfgang Stephan in 2013, and a single line of *D. bipectinata* from the former UCSD Species Stock Center (stock 0000-1029.01) that was collected by Artyom Kopp. We also sequenced a single *Sxl* allele from *D. guanche* (obtained from the former *Drosophila* Species Stock Center at University of California San Diego; stock number 14011-0095.01) that was collected in the Canary Islands, Spain by Nicolas Gompel, and *D. atripex* (stock number V251 provided to us by Artyom Kopp and originally collected in Kuching, Malaysia in 1979 by Fuyama, Hihara, and Watanabe), which were used for analyses that require divergence for the *D. pseudoobscura* and *D. ananassae* datasets, respectively. Our sequences are available via Genbank accession numbers KT935592–KT935663, MZ269293, MZ269294, MZ269302, and MZ269303. All other sequences used in our analyses were those from *Drosophila* 12 Genomes Consortium *et al.* (2007) or Chen *et al.* (2014) and downloaded from Flybase. We do not feel that differences in strain backgrounds, provenance and type (isofemale *vs* extracted X chromosome stock) would have biased our findings in any significant way. In fact, the lines have been in the lab long enough and maintained as vial cultures that most are essentially inbred lines fixed for a single allele which simplifies the base calling in sequencing.

The following triplets of species were independently aligned using MegAlign (DNASTAR Inc., Madison WI): *D. melanogaster*, *D. simulans*, *D. yakuba*; *D. ananassae*, *D. atripex*, *D. bipectinata*; and *D. pseudoobscura*, *D. miranda*, *D. guanche*. Gaps within coding regions were manually adjusted to ensure the sequences remained in-frame. Indels were in multiples of three nucleotides and the manual adjustment of the alignment ensured that triplet indels delete or add an encoded amino acid. Flanking amino acids were used to determine which amino acid was deleted or added. Population genetic analyses were performed using DnaSP 5.10.1 (Librado and Rozas 2009). When using coalescent simulations to obtain the *P*-values of site frequency-based tests we incorporated recombination rate estimates (following Przeworski *et al.* 2001). For the *D. melanogaster* and *D. simulans* datasets, we used the *D. melanogaster* Recombination Rate Calculator (Comeron *et al.* 2012) estimate of *r* = 3.34 × 10^−8^ recombinants per base-pair per generation for the *Sxl* region of the X chromosome. This translates to an estimated *R* = 152 for the 1500 base pair region sequenced (*R* = 3N_e_r since *Sxl* is X-linked and assuming a population size of 1.0 × 10^6^). For *D. ananassae* and *D. pseudoobscura*, the values of *R* were estimated from the polymorphism data directly using DNAsp 5.10.1 (Librado and Rozas 2009) as *R* = 194 and *R* = 6, respectively.

For *D. melanogaster*, we also incorporated demography into our neutral simulations when obtaining our significance cutoffs. These simulations were done using the program msABC (Pavlidis *et al*. 2010 ). There is growing evidence that African populations of *D. melanogaster* have experienced changes in effective population size over time (Glinka *et al*. 2003; Li and Stephan 2005; Hutter *et al.* 2007; Haddrill *et al*. 2008 ; Duchen *et al* 2013; ). However, given the large effective population size of these species and signatures of a high rate of adaptation (*e.g.*, Begun *et al*. 2007; Langley *et al*. 2012), inferring demographic parameters is challenging. Because of this, we simulated three different scenarios: standard neutral equilibrium model, standard neutral with exponential growth as estimated by Hutter *et al.* (2007), and standard neutral with a three phase bottleneck as estimate by Duchen *et al.* (2013). We supplied msABC with uniform prior distributions for theta and all demographic parameters. The prior distribution for theta for *D. melanogaster* was obtained from Pool *et al.* (2012) and ranged between 0.006 and 0.009 per site. The resulting *P*-values are the proportion of simulated datasets that were less than (for negative statistics) or greater than (for positive statistics) our observed test statistic for *Sxl*. The *P*-values were adjusted for multiple testing following the Bonferroni method.

The McDonald-Kreitman test (MKT) was done manually following the method’s original implementation (McDonald and Kreitman 1991) by combining polymorphism from multiple species if it was available. If a position in the alignment had more than one nucleotide segregating within a species’ population sample, it was labeled as polymorphic. Divergent sites were those for which all alleles from one species differed from all the alleles of the other two species.

To test for evidence of departures from neutrality for synonymous sites at *Sxl*, we used the method of Bauer DuMont *et al.* (2004). This method looks for differences in the rates of preferred and unpreferred codon substitutions per site in a manner similar to a dN/dS comparison (Nei and Gojobori 1986). Statistical significance is assessed by a 2 × 2 contingency table comparison.

In order to test for evidence of departures from selective neutrality in rates and patterns of sequence evolution at *Sxl* across a broader group of *Drosophila* species, we first retrieved the *Sxl* gene region sequences from FlyBase for the following 20 *Drosophila* species: *D. melanogaster*, *D. sechellia*, *D. yakuba*, *D. erecta*, *D. eugracilis*, *D. ficusphila*, *D. rhopaloa*, *D. elegans*, *D. takahashii*, *D. biarmipes*, *D. kikkawae*, *D. bipectinata*, *D. ananassae*, *D. miranda*, *D. pseudoobsura*, *D. persimilis*, *D. willistoni*, *D. mojavensis*, *D. virilis*, and *D. grimshawi*. *Sxl* is an alternatively spliced locus. To ensure we are analyzing orthologous exons, we first made alignments of the entire gene region (introns and exons) using the web-based versions of the alignment programs Muscle (Edgar 2004) (http://www.ebi.ac.uk/Tools/msa/muscle/). We then used the annotated exons of *D. melanogaster* as a guide to identify orthologous coding sequences (CDS) for *Sxl’s* Isoform L (6 exons—the female-specific splice variant; Bell *et al.* 1988) from each aligned sequence. This isoform contains the poly-proline region of the Sxl protein where the female-sterile *Sxl* variants are located (Starr and Cline 2002).

We estimated a *Sxl* gene tree across these 20 *Drosophila* species using Mega 5.1 (Tamura *et al.* 2011). A maximum likelihood tree was estimated using all nucleotide sites, default parameters, and the GTR substitution model with gamma-distributed site variation. To test for selection across the estimated *Sxl* tree we used Hyphy (Kosakovsky Pond *et al.* 2005) run online using the DataMonkey website (http://www.datamonkey.org/). A model selection procedure was conducted to determine that the best nucleotide substitution model for the data was TrN93 (Tamura and Nei 1993) which was used in all subsequent analyzes. We ran GARD (Kosakovsky Pond *et al.* 2006a, 2006b) to look for evidence of recombination across these species at *Sxl* (that would reflect incomplete ancestral polymorphism sorting) using a general discrete site-to-site rate variation and three rate classes. To detect evidence of purifying and/or diversifying selection across sites and lineages we used the following Hyphy programs: BranchRel, GAbranch, FUBAR, and MEME.

## Data availability

DNA sequences generated as part of this study are available from Genbank with accession numbers: KT935592-KT935663, MZ269293, MZ269294, MZ269302, and MZ269303.

## Results

We surveyed DNA variability at the population level at *Sxl* for four species of *Drosophila* (*D. melanogaster*, *D. simulans*, *D. ananassae*, and *D. pseudoobscura*), the first three of which have evidence of *W. pipientis* infection (Mateos *et al.* 2006). Even though 37 lines of *D. pseudoobscura* were surveyed for infection, none were found to be infected with *W. pipientis* (Mateos *et al.* 2006).

We find that Sxl is a very conserved protein with little to no nonsynonymous polymorphism or divergence within or between the 20 *Drosophila* species surveyed (Table 1). In addition, levels of synonymous polymorphism and divergence between *D. melanogaster* and *D. simulans* are below the average reported by Andolfatto (2005). The same is true for synonymous variation observed within and between *D. pseudoobscura* and *D. miranda* as compared to that reported by Haddrill *et al.* (2010). While synonymous polymorphism is slightly lower at *Sxl* within *D. ananassae* the level of divergence between *D. ananassae* and *D. atripex* is similar to values previously reported (Grath *et al.* 2009; Choi and Aquadro 2014).

**Table 1:** Levels of within-species variation and divergence between *Drosophila* species and results of tests to detect departures from a neutral site frequency spectrum

	θ	π	Div^c^	Taj-D^a^ (*P*-value^b^)	FW-H^a^ (*P*-value^b^)
*D. melanogaster* (*n* = 20)					
Synonymous	0.012	0.011	0.069	−0.267	−2.26
Nonsynonymous	0	0	0	(0.21; 0.80; 0.70)	(**<0.0002; <0.0002; <0.0002**)
Intron	0.019	0.019	0.083		
*D. simulans* (*n* = 10)					
Synonymous	0.017	0.013	0.069	−1.045	3.47
Nonsynonymous	0	0	0	(**0.001**)	(0.87)
Intron	0.031	0.025	0.083		
*D. ananassae* (*n* = 13)					
Synonymous	0.010	0.010	0.209	0.349	−4.333
Nonsynonymous	0.000	0.000	0.003	(0.166)	(0.038)
Intron	0.021	0.025	0.299		
*D. pseudoobscura* (*n* = 29)					
Synonymous	0.011	0.007	0.073	−1.293	−0.539
Nonsynonymous	0.0004	0.0001	0.002	(0.038)	(0.311)
Intron	0.014	0.008	0.111		

^a^ Test statistic when using all sites in the analysis: Tajima’s D (Taj-D) and Fay and Wu’s H (FW-H).

^b^ Proportion of simulated datasets that were equal to or less than (for negative statistics) or equal to or greater than (for positive statistics) our observed test statistic for *Sxl.* These are two-sided tests, for which we do two tests per species resulting in a significant cutoff level of 0.0125 (0.025/2). For *D. melanogaster*, the *P*-value was calculated by simulating different demographic scenarios listed in this order in parenthesis: Standard neutral; Bottleneck with exponential growth and a 3 Epoch bottleneck (as described in Materials and Methods). *P*-values significant after multiple testing corrections in bold.

^c^Uncorrected divergence between the following species pairs: *D. melanogaster* and *D. simulans*; *D. ananassae* and *D. atripex*; and *D. pseudoobscura* and D. miranda.

Previous studies have reported a skew in the Site Frequency Spectrum (SFS) toward rare alleles, as illustrated by a general negative Tajima *D* (Tajima 1989) test statistic, in all of the species included in our study ( Kliman *et al.* 2000; Machado *et al.* 2002; Das *et al.* 2004; Andolfatto 2007; Grath *et al.* 2009; Haddrill *et al.* 2010; Jensen and Bachtrog 2011). At *Sxl*, we observe a negative Tajima *D* for all species, except *D. ananassae*. Tajima *D* in *D. simulans* rejects the hypothesis of a SFS at equilibrium with 67% (33/49) of the polymorphisms being singletons. These singletons are evenly distributed across synonymous (seven singleton/10 total) and intron (26 singleton/39 total) sites. At *Sxl*, Fay and Wu’s *H* is negative in all species but *D. simulans*. Fay and Wu’s *H* statistic is significantly negative in *D. melanogaster* even when considering two different demographic scenarios estimated for African populations of this species (Hutter *et al.* 2007; Duchen *et al.* 2013).

The MKT (McDonald and Kreitman 1991) is used to detect departures from the neutral expectation that synonymous and nonsynonymous variants will have similar ratios of within to between species variation. A rejection in the direction of an excess of nonsynonymous divergence is typically interpreted as evidence of repeated amino acid substitutions due to positive selection. As seen in Table 1, there are few nonsynonymous changes at *Sxl*. The MKT does not reject neutral expectations when *D. ananassae* and *D pseudoobscura* polymorphism is compared to divergence to *D. atripex* and *D. miranda*, respectively (Table 2). We observe no amino acid differences within or between *D. melanogaster* and *D. simulans*. However, when considering the *Sxl* sequence from two additional and closely related Drosophila species, *D. yakuba* and *D. erecta*, we observe seven amino acid substitutions along the lineage leading to *D. melanogaster* and *D. simulans*. Given this observation, we chose to apply the MKT to these species in a manner similar to its first implementation (McDonald and Kreitman 1991) by combining the polymorphism from *D. melanogaster* and *D. simulans*. Our divergent changes in this MKT comparison are all differences since the most recent common ancestor of the *D. melanogaster* and *D. simulans* lineage rooted by the *D. yakuba* and *D. erecta* lineages. This combined polymorphism MKT rejects neutral expectations, in the direction suggestive of an excess of amino acid substitutions.

**Table 2:** Results of the McDonald-Kreitman test at *Sxl* between three different sets of *Drosophila* species

	Synonymous	Nonsynonymous	*P*-value^a^
Single species polymorphism			
*D. ananassae*/*D. atripex*			
Polymorphic	8	0	
Fixed divergent	44	2	1.00
*D. pseudoobscura*/*D. miranda*			
Polymorphic	9	1	
Fixed divergent	14	0	0.417
Two species polymorphism combined			
*(D. melanogaster ± D. simulans)*/*D. yakuba*			
Polymorphic	19	0	
Fixed divergent	19	7	**0.015**

^a^ Fisher exact test *P*-value (significant P values indicated by bold for P<0.05)

The significant MKT for *D. melanogaster*/*D. simulans* could be due to selective fixation of amino acid differences or to selection acting on synonymous changes. While synonymous sites have traditionally been assumed as the neutral yardstick of molecular evolution, there is evidence that this assumption may be invalid, for at least some genes in *Drosophila* (*e.g.*, Akashi and Schaeffer 1997; Bauer DuMont *et al.*^2004^, ^2009^; Poh *et al.* 2012; Lawrie *et al.* 2013). We looked for evidence of selection acting on synonymous changes at *Sxl* using a per site counting method (CF-test) similar to a dN/dS comparison (Bauer DuMont *et al.* 2004), except in this test we are comparing the number of changes toward unpreferred or preferred codons per the number of unpreferred and preferred “sites”. Along the *D. simulans*, *D. ananassae*, and *D. pseudoobscura* lineages we observe a significant departure from neutrality in the direction suggesting a selective advantage of mutations toward preferred codons at *Sxl* (Table 3). The test did not reject in *D. melanogaster*.

**Table 3:** Results of the CF Test at *Sxl* along four *Drosophila* lineages

	Unpreferred	Preferred	*P*-value^a^	Direction of departure
*D. melanogaster* lineage				
Fixed substitutions	5	0		
Sites	150	31	0.303	No preference
*D. simulans* lineage				
Fixed substitutions	1	2		
Sites	150	31	0.022	Preferred codons favored
*D. ananassae* lineage				
Fixed substitutions	6	11		
Sites	174	29	<0.001	Preferred codons favored
*D. pseudoobscura* lineage				
Fixed substitutions	1	3		
Sites	154	31	0.002	Preferred codons favored

^a^ Fisher exact test *P*-value.

Selection acting on synonymous sites can lead to false positive MKT results by elevating the synonymous polymorphism cell of the 2 × 2 table, due to segregating slightly deleterious synonymous variants. One method proposed to mitigate the effects of selective constraint on the MKT is to remove low frequency polymorphisms (frequency less than 15%) from the analysis (Fay *et al.* 2001). Thus, given the CF-test results for *D. simulans*, we also carried out the MKT by first removing two derived unpreferred polymorphic singletons each with a frequency of 10% in sample in *D. simulans*. The *D. melanogaster*/*D. simulans* MKT remains significant (*P*-value = 0.031).

**Figure 1:**
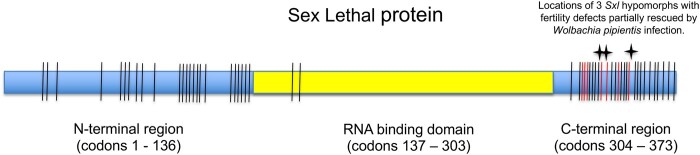
Schematic of region of the Sxl protein studied (exons 5–8 of the female transcript; Bell et al. 1988). Intron/exon boundaries were conserved across the species studied consistent with Zhang et al. (2014). The yellow box denotes the location of the RNA binding domain of the Sxl protein, the rest of which is indicated by the blue boxes. Vertical lines show the locations of all amino acid substitutions at *Sxl* across 20 *Drosophila* species with the red lines being those that have occurred specifically on the lineage leading to *D. melanogaster* and *D. sechellia*. Stars at C-terminal end of protein denote the relative location of the mutations that generate the mutant alleles shown to interact genetically with *W. pipientis*.

**Figure 2:**
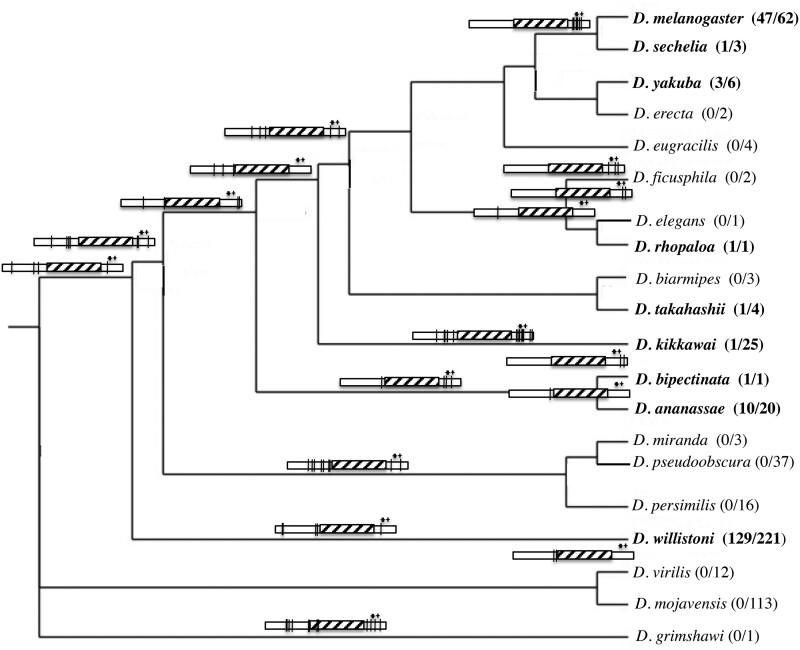
*Sxl* gene tree schematically showing the branches for which amino acid substitutions have occurred. The rectangles denote the Sxl protein with the vertical black lines indicating the location of the amino acid change(s) along that lineage. Hatched box denotes the location of the RNA binding domains of the Sxl protein. Stars at C-terminal end of protein denote the relative location of the three mutations that generate the mutant alleles shown to interact genetically with *W. pipientis*. Species names in bold indicate *W. pipientis* has been detected in that species with the numbers of *W. pipientis* positive lines relative the total number of lines screened given in parenthesis. *W. pipientis* data for all species are from Mateos et al. (2006), as well as for *D. erecta* (Zabalou 2004), *D. kikkawai* (Bennett et al. 2012), *D. bipectinata* (Ravikumar et al. 2011), and *D. willistoni* (Muller et al. 2013).

To further assess the molecular evolution at *Sxl* we made a CDS alignment using the program Muscle for *Sxl’s* Isoform L (all 6 exons of the female-specific splice variant including exons 5–8 for which we assessed polymorphism; Bell *et al.* 1988, which are exons 3–8 of the flybase.org Sxl annotation), and exons 5–10 described by (Samuels *et al*. 1991) for the following 20 Drosophila species: *D. melanogaster*, *D. sechellia*, *D. yakuba*, *D. erecta*, *D. eugracilis*, *D. ficusphila*, *D. rhopaloa*, *D. elegans*, *D. takahashii*, *D. biarmipes*, *D. kikkawae*, *D. bipectinata*, *D. ananassae*, *D. miranda*, *D. pseudoobsura*, *D. persimilis*, *D. willistoni*, *D. mojavensis*, *D. virilis*, and *D. grimshawi*. The intron-exon boundaries for the six exons are conserved for these taxa sufficiently for coding sequence alignments to be made with confidence (consistent with Zhang *et al.* 2014). We observe 86 amino acid substitutions at 53 codon positions among these species for the six exons of isoform L of *Sxl*. Roughly 38% of the codons that have experienced an amino acid substitution have been hit multiple times (20/53; note that some multiply hit amino acid positions had more than three different amino acids segregating among the species). The conservation of the RNA binding domain of the Sxl protein has been previously noted (Zhang *et al.* 2014). In agreement, 84 out of the 86 amino acid changes occurred outside the RNA binding domain region, between codons 1–136 and 304–373 in our alignment (Figures 1 and 2). We will call these non-RNA binding regions of the six exon region of the Sxl protein the N-terminal and C-terminal regions, respectively. The amino acid substitutions at Sxl have not occurred equally between the N-terminal and C-terminal regions after taking into account their differences in total codon length. We observe significantly more amino acid substitutions in the C-terminal region (24 codons with an amino acid substitution out of 137 total codons in N-terminal region versus 27 substituted codons out of 70 total codons in C-terminal region; 2 × 2 table chi-square = 11.1, *P*-value = 0.001). The two regions have experienced a similar proportion of multiple hit codons (10 multiple hit codons out of 24 total codons with an amino acid substitution in N-terminal region versus 10 out of 27 such codons in C-terminal region).

To determine whether the apparent heterogeneity in amino acid substitution at *Sxl* showed evidence resulting from positive selection, we analyzed the data using the phylogeny-based Hyphy method (Kosakovsky Pond *et al.* 2005; Delport *et al.* 2010). Phylogenetic incongruence between species along a gene sequence, due to sorting of ancestral polymorphisms, can have adverse effects on phylogenetic-based inferences of positive selection (Wong *et al.* 2007). We first performed the Hyphy GARD method (Kosakovsky Pond *et al.* 2006b), which is designed to detect such incongruences. None were detected at a *P*-value cutoff of 0.10. Therefore, we used a maximum-likelihood gene tree, made from 3rd codon positions, in subsequent analyzes.

We applied the following methods of the Hyphy package to our data: GAbranch (Kosakovsky Pond and Frost 2005), BranchRel (Kosakovsky Pond *et al.* 2011), MEME (Murrell *et al.* 2012), and FUBAR (Murrell *et al*. 2013). GAbranch detects significant heterogeneity across the *Sxl* phylogeny in the rate of nonsynonymous compared to synonymous evolution (the dN/dS ratio). The best fitting model includes three rate classes, yet the dN/dS for the highest class is only 0.115 across the *Sxl* locus. The posterior probabilities suggest that the following branches are within the highest dN/dS rate class and that they are evolving at a significantly different rate than other branches in the tree: the branch leading to *D. melanogaster* and *D. sechellia*, the branch leading to *D. elegans* and *D. rhopaloa*, the branch leading to the melanogaster species group, and the *D. kikkawei* lineage (Figure 2).

The BranchRel method pools information across sites to estimate selection parameters along branches. The method reports the proportion of codons along each lineage that have evolved under three selection regimes: negative selection, neutral/nearly neutral or episodic positive selection. BranchRel confirmed ubiquitous evidence of amino acid constraint (negative selection) across the *Sxl* phylogeny. After multiple testing corrections, no lineage has significant evidence of positive diversifying selection. Similar results were obtained using MEME.

FUBAR is used to detect selection pressure acting on individual codons. The strength of this method is that it does not restrict the parameter space for which nonsynonymous and synonymous rates are drawn from during the maximum likelihood process. FUBAR does not detect any sites under diversifying selection with a posterior probability greater than 0.90. However, it does detect 258 codons under negative selection with a posterior probability greater than 0.90, which is roughly 70% of the protein. Just over half of these negatively selected sites (140) are located within the RNA binding domain. We observe no significant difference in the number of negatively selected sites between the N-terminal and C-terminal regions of *Sxl* relative to their respective lengths (N-terminal: 77 negative selective sites in 137 codons versus C-terminal: 41 negative selected sites in 70 codons; 2 × 2 chi-square = 0.023, *P*-value = 0.879).

Interestingly, as illustrated in Figure 2, the seven amino acid differences observed on the lineage leading to the ancestor of the *D. melanogaster* and *D. simulans* species group (with which we observe the significant MKT), cluster with the location of the mutations previously shown to genetically interact with *W. pipientis* (Starr and Cline, 2002; Sun and Cline 2009).

## Discussion

In this study, we use population and phylogenetic based methods to examine the molecular population genetics and evolution of the *Sxl* locus within the genus *Drosophila*, for which *Sxl* is the master switch in sex-determination. We were motivated by the observation of a genetic interaction between *W. pipientis* infection and some mutant alleles at *Sxl* in *D. melanogaster* (Starr and Cline 2002; Sun and Cline 2009)*.* It is not known if this interaction is due to a ubiquitous effect of *W. pipientis* on overall egg production, or if it is due to a direct interaction between the endosymbiont and the *Sxl* locus or protein product.

Considering polymorphism data alone, we detect patterns consistent with a recent selective sweep in both *D. simulans* and *D. melanogaster* with the Tajima *D* and Fay and Wu *H* tests, respectively. These significant skews in the frequency spectrum in these species could be due to a variety of evolutionary forces including demography, a selective sweep associated with the fixation of a linked positively selected mutation, or segregating weakly deleterious mutations. *D. simulans* is thought to have experienced a recent population expansion resulting in a general tendency for loci in this species to have negative Tajima *D* test statistics (Kliman *et al.* 2000). The significant Fay and Wu’s *H* test in *D. melanogaster* remains significant even when demography is incorporated into the null distribution of the test, suggesting that in this species we are detecting a recent selective sweep. These signatures of positive selection would not be due to an amino acid fixation, given that there are no amino acid differences between *D. melanogaster* and *D. simulans* at *Sxl.*

*Sxl’s* long-term evolution within *Drosophila* largely reflects strong conservation of protein sequence, as noted previously by Mullon *et al.* (2012). We detect the action of negative selection both along lineages and at specific codons. The FUBAR method estimates that 70% of the *Sxl* codons are selectively constrained, suggesting that negative selection has had a pervasive effect on *Sxl’s* molecular evolution. However, we do observe amino acid differences across these *Drosophila* species and the pattern of these substitutions is heterogeneous. For example, GA-branch method detects significant variation in the dN/dS ratio across the *Drosophila* species included in this analysis. This heterogeneity at *Sxl* could be due to sporadic relaxations of the negative selection that dominates *Sxl’s* molecular evolution or due to sporadic bursts of positive selection.

The Hyphy methods used to detect recurring or episodic positive selection fail to do so after multiple testing corrections. However, we note that the pervasive negative selection observed at *Sxl* could confound these methods, especially if the positive selection is weak or if only a few sites are affected (Kosakovsky Pond *et al.* 2011). Our current data shows no evidence of long-term or recent positive selection at *Sxl* along the *D. ananassae* and *D pseudoobscura* lineages. In contrast, there are weak signatures of both types of selection in *D. melanogaster* and *D. simulans*, so we focus on the molecular evolution of these species and the lineage leading to their common ancestor.

The lineage leading to *D. melanogaster and D. simulans* shows a decoupling of synonymous and nonsynonymous evolution with the MKT in the direction of an excess of nonsynonymous divergence. This result is due to seven amino acid substitutions on the lineage leading to the *D. melanogaster*/*D. simulans* clade. These nonsynonymous substitutions cluster with the locations of the *Sxl* alleles that genetically interact with *W. pipientis.* In addition, this region of the Sxl protein (the C-terminal non-RNA binding region) has experienced significantly more amino acid substitutions than the N-terminal region. This elevation in amino acid substitutions does not appear to be due to a simple relaxation of constraint as we observe no difference between the C- and N-terminal regions in the number of codons predicted to be experiencing negative selection.

These results are suggestive of positive selection being at least partially responsible for the fixations of these seven *D. melanogaster*/*D. simulans* amino acid substitutions. However, there are other possible explanations for these results such as synonymous site evolution and/or changes in effective population. The seven amino acid fixations could be due to the fixation of slightly deleterious mutations if the effective population size was smaller on the branch leading to the *D. melanogaster*/*D. simulans*. However, the relaxation of constraint is expected to affect both synonymous and nonsynonymous substitutions. We assume the ancestral state in codon preference is toward preferred synonymous codons at *Sxl*, given the results of the CF-test. If relaxation of constraint were responsible for the burst of amino acid fixations on the *D. melanogaster*/*D. simulans* lineage we may also expect a burst of derived unpreferred substitutions, but we do not observe this. We observe an equal number of preferred and unpreferrred changes on this lineage (data not shown).

Infection dynamics of *W. pipientis* appear to be sporadic and variable both between and within lineages, with uninfected species interspersed with infected species throughout the phylogeny (*e.g.*, Mateos *et al.* 2006) consistent with multiple losses or gains of infection. The resulting uncertainty in the infection history of species unfortunately prevents us from reliably testing for correlations between *W. pipientis* infection status and burst of positive selection; there are many factors that could weaken our ability to detect an association.

In this study, we present data revealing that the fixation of amino acid variants at Sxl appears to be heterogeneous across the region of the major transcript that we studied, potentially weakening phylogenetic methods to detect positive selection or associations with character states. The MKT does reject neutrality a manner suggestive of an acceleration of nonsynonymous fixations. Interestingly, these two observations of sequence evolution are quite similar to what we have previously found for the *bag of marbles* (*bam*) gene (Bauer DuMont *et al.* 2007, Flores *et al.* 2015a), which like *Sxl* shows a partial rescue of fertility defects in a hypomorph allele by infection with *W. pipientis* (Flores *et al.* 2015b). And while *Sxl* has an important function in sex determination in *Drosophila* development (Bell *et al.* 1988) it also plays a critical role, along with *bag of marbles* (*bam*), in the maturation of cystoblasts during oogenesis (Chau *et al.* 2009). Nonetheless, the extent of amino acid differences is very different between these loci with there being 59 fixed amino acid substitutions between *D. melanogaster* and *D. simulans* at *bam* and none at *Sxl*.

Our results do not allow us to draw strong conclusions regarding the role of positive selection on the molecular evolution of the *Sxl* locus within *Drosophila*, but they also do not allow us to discount the influence of both long term (MKT) or recent (Tajima D and Fay and Wu H tests) positive selection in *D. melanogaster* and *D. simulans*. Current data and methodology also do not allow us to make a direct connection between *W. pipientis* infection and selective pressures acting on *Sxl* or *bam*. So, it remains open whether the genetic interaction between mutant *Sxl* and *bam* alleles and *W. pipientis* is due to a direct interaction between Sxl and Bam proteins and this endosymbiont. Our results do motivate screening for genetic interactions between *W. pipientis* and other mutant alleles at *Sxl* and other GSC loci because the observation that *W. pipientis* rescues some but not other mutations (*e.g.*, Starr and Cline 2002) will help refine candidates for the mechanism(s) by which *W. pipientis* is manipulating *Drosophila* reproduction.
